# Maternal and neonatal IgG against *Klebsiella pneumoniae* are associated with lower risk of neonatal sepsis: A case-control study of hospitalized neonates in Botswana

**DOI:** 10.1371/journal.pgph.0003350

**Published:** 2024-12-05

**Authors:** Siqi Linsey Zhang, Carolyn M. McGann, Tereza Duranova, Jonathan Strysko, Andrew P. Steenhoff, Alemayehu Gezmu, Britt Nakstad, Tonya Arscott-Mills, One Bayani, Banno Moorad, Nametso Tlhako, Melissa Richard-Greenblatt, Weiming Hu, Paul J. Planet, Susan E. Coffin, Michael A. Silverman

**Affiliations:** 1 Division of Infectious Diseases, Children’s Hospital of Philadelphia, Philadelphia, Pennsylvania, United States of America; 2 Division of Neonatology, Children’s Hospital of Philadelphia, Philadelphia, Pennsylvania, United States of America; 3 Perelman School of Medicine, University of Pennsylvania, Philadelphia, Pennsylvania, United States of America; 4 Department of Paediatric & Adolescent Health, Faculties of Medicine & Health Sciences, University of Botswana, Gaborone, Botswana; 5 Botswana-University of Pennsylvania Partnership, Gaborone, Botswana; 6 Department of Pediatrics, Children’s Hospital of Philadelphia, Philadelphia, Pennsylvania, United States of America; 7 Hospital for Sick Children, Toronto, Canada; 8 Department of Laboratory and Pathobiology, University of Toronto, Toronto, Canada; 9 Division of Gastroenterology, Hepatology and Nutrition Children’s Hospital of Philadelphia, Philadelphia, Pennsylvania, United States of America; 10 CHOP Microbiome Center, Children’s Hospital of Philadelphia, Philadelphia, Pennsylvania, United States of America; Simon Fraser University, CANADA

## Abstract

Sepsis is the leading postnatal cause of neonatal mortality worldwide. Globally *Klebsiella pneumoniae* is the leading cause of sepsis in hospitalized neonates. This study reports the development and evaluation of an ELISA for anti-*Klebsiella* IgG using dried blood spot (DBS) samples and evaluates the association of anti-*Klebsiella* IgG (anti-Kleb IgG) antibodies in maternal and neonatal samples with the risk of neonatal sepsis. Neonates and their mothers were enrolled at 0–96 hours of life in the neonatal unit of a tertiary referral hospital in Gaborone, Botswana and followed until death or discharge to assess for episodes of blood culture-confirmed neonatal sepsis. Neonates with sepsis had significantly lower levels of *Kleb-*IgG compared to neonates who did not develop sepsis (Mann-Whitney U, p = 0.012). Similarly, samples from mothers of neonates who developed sepsis tended to have less *Kleb-*IgG compared to mothers of controls. The inverse correlation between *Kleb-IgG* levels and all-cause bacteremia suggests that maternal *Kleb-*IgG may be protective through cross-reactivity with common bacterial epitopes. These data support the continued use of immunoglobulin assays using DBS samples to explore the role of passive immunity on neonatal sepsis risk and reaffirm the critical need for research supporting the development of maternal vaccines for neonatal sepsis.

## Introduction

Sepsis is the leading postnatal cause of neonatal deaths, accounting for nearly one million deaths annually [1]. The incidence, causative pathogens and mortality attributable to neonatal sepsis vary globally [2]. In high-income countries, the most common causes of early-onset sepsis (EOS) are Group B streptococcus (GBS) and *Escherichia coli* whereas the vast majority of late-onset sepsis (LOS) cases are caused by Gram-positive organisms such as coagulase-negative staphylococci (CoNS) and *Staphylococcus aureus* [3–5]. In contrast, both EOS and LOS are predominantly caused by Gram-negative organisms in low- and middle-income countries (LMIC) [6–8]. In Botswana and other sub-Saharan African countries, Gram-negative sepsis accounts for about 60% of neonatal sepsis, with a predominance of *Klebsiella* spp. [6, 9, 10] Notably, over three-quarters of the global neonatal sepsis deaths occur in LMIC signifying the critical unmet need to better understand the immune system features that predispose to, or protect against neonatal sepsis in these settings [11]. There has been a global focus on strategies to prevent neonatal infection, including prophylactic antibiotics and novel vaccine development for pathogens including *Klebsiella pneumoniae*; however, geographic variation in the causes of neonatal infections highlights the need to better understand the risk factors for Gram-negative sepsis in neonates.

Since neonatal innate and adaptive immune systems are immature at birth, the immunoglobulins obtained through transplacental transfer and breastmilk provide critical protection from sepsis [12–15]. Immunoglobulin G (IgG) is the primary antibody transplacentally transferred and plays an essential role in protecting neonates from infection [16, 17]. Although the transfer of IgG begins at 13 weeks of gestation, it accelerates significantly after 36 weeks. Thus preterm, as compared to term, neonates are less protected by passive immunity contributing to their higher risk of sepsis [17, 18].

We hypothesized that lack of maternal IgG antibodies that target specific pathogenic bacteria may predispose neonates to develop sepsis. We developed an enzyme-linked immunosorbent assay (ELISA) to evaluate the degree to which the risk of neonatal sepsis is associated with the level of IgG from maternal and neonatal serum capable of binding to *K*. *pneumoniae* (*Kleb-*IgG).

## Methods

### Setting and population

This study used samples collected from a cohort of 467 neonates admitted to the neonatal unit at a tertiary referral hospital in Gaborone, Botswana from November 1, 2020 –December 31, 2021. Participants were eligible for enrollment in the parent study if admitted to the neonatal unit within the first 3 days of life and were followed until discharge or death. A research nurse approached mothers after delivery to obtain written informed consent in Setswana or English. The study was approved by the institutional review boards of the Botswana Ministry of Health Research and Development Committee (protocol 13/18/1, Jan. 16, 2020), the University of Botswana (protocol 147, Dec. 13, 2019), the University of Pennsylvania (protocol 833786, April 22, 2020), the hospital IRB (protocol 708, April 2, 2020) and Children’s Hospital of Philadelphia (protocol 19–016848, July 25, 2020). Cases of sepsis were matched with controls admitted to the neonatal unit but without sepsis in a 1:4 ratio based on the nearest sample collection date and gestational age (GA). Subjects unable to be matched by GA were matched with subjects with the nearest GA. Subjects with missing data or samples were excluded. Patients and the public were not included in the design or implementation of this study. Five patients with mothers under the age of 18 were unintentionally enrolled. This protocol deviation was reported to all IRBs and these patients’ data were excluded.

### Sample collection

Dried blood spots (DBS) were collected by heel stick from enrolled neonates and finger stick from mothers timed with a clinically indicated blood sample and stored on filter paper (Whatman 903, Cytiva, USA). We were unable to stick a second time if a patient stopped bleeding, thus, collected volumes varied. Blood spots were dried at room temperature for three hours and placed in plastic bags with desiccant packs. They were stored at room temperature before shipment (13–26°C) and at -20°C after shipment from Botswana to the United States to enhance the stability of the samples. Samples were transported at room temperature and contained both neonatal and maternal DBS collected within 4 months of delivery to the US. Sample integrity was established prior to analysis.

Blood cultures were drawn at the discretion of the clinical team for suspected sepsis using conventional clinical criteria [19, 20]. Samples were incubated on an automated blood culture system (BACT/ALERT, bioMerieux, Marcy l’Etoile, France) for up to 5 days or until blood culture positivity. Neonatal sepsis was defined as a positive blood culture. CoNS spp. and other skin commensals were deemed contaminants unless ≥2 cultures within 7 days grew this organism [21].

### Extraction of antibodies from dried blood spots

To extract antibodies from DBS, a 6.35-mm-diameter punch from a saturated blood spot was placed in an Eppendorf tube with 500 μl of elution buffer (phosphate buffered saline [PBS] with 0.1% BSA and protease inhibitor cocktail (Roche, catalog no. 04693159001) then was incubated overnight on a shaker at 4°C. Samples were then centrifuged for 5 mins at 8,000g at 4°C to pellet debris and the supernatants were collected and stored at -20°C.

### Immunoglobulin quantification for DBS eluates

To quantify the total amount of IgG and IgA present in each eluate, ELISA was performed using Bethyl Human ELISA Quantitation Set (E80-100, E80-102, E80-104) per manufacturer instructions (Bethyl Laboratories, Montgomery, TX, USA).

### Whole genome sequencing

A *K*. *pneumoniae* isolate was obtained from a blood culture sample from a hospitalized neonate at the study site in Botswana during the study period. To further characterize this strain, we performed whole genome sequencing on this isolate on the Illumina NovaSeq platform using a 300-cycle kit which generated 10,854,787 150bp paired-end reads. The Sunbeam pipeline was used to ensure that there was no human DNA contamination [22]. The resulting reads were assembled using SPAdes v4.0.0 (Using SPAdes De Novo Assembler). The quality of the genome was assessed using CheckM and Mash [23, 24]. Sequence type and putative *Klebsiella* genes were identified by using KleborateV2.4.1 [25]. The sequencing data was deposited to the Sequence Read Archive (SRA) under the accession number PRJNA1140575.

### Bacterial-binding IgG quantification for DBS eluates using ELISA

We developed an ELISA using bacteria-coated plates to determine the degree to which neonatal and maternal antibodies bind to bacterial surface epitopes of five different *K*. *pneumoniae* isolates, including four blood culture isolates and one environmental *K*. *pneumoniae* isolate from a hospital sink. We noted similar binding of each serum IgG antibodies sample to the isolates (**S1 Fig**) and thus focused upon MF391, a neonatal bloodstream isolate.

For the ELISA, the MF391 *K*. *pneumoniae* isolate was subcultured to a Luria broth (LB) solid media plate and incubated at 37°C overnight. A single colony was selected and transferred into 5 mL of LB to establish a liquid culture. The following day, the culture was centrifuged (10 minutes at 4,000xg), fixed with 4% paraformaldehyde for 20 minutes and washed three times in PBS (pH 7.4). The pellet was resuspended in PBS and diluted to achieve an optical density of 1.0. A 100 μl volume of bacteria was then added to each well of a 96-well ELISA plate (Thermo Scientific Nunc, 442404). The plate was incubated overnight at 4°C, then washed (0.07 M NaCl and 0.025% Tween 20 dissolved in Tris buffer) and blocked (0.007 M NaCl and 1% BSA in Tris) for 30 minutes at room temperature. DBS eluates were diluted (0.007 M NaCl, 1% BSA, 0.025% Tween 20 in Tris buffer) to a uniform IgG concentration of 0.4 ng/μl. 100 μl of the diluted DBS samples were added to each well and incubated overnight at 4°C. The wells were then washed and incubated with horseradish peroxidase-conjugated anti-IgG antibody (1 mg/mL diluted 1:150,000) for an hour and developed for 15 minutes in 3,3’,5,5’-tetramethylbenzidine with 0.18M H_2_SO_4_ as the stopping reagent. The absorbance at 450nm was measured immediately after adding the stopping reagent on an Enspire Multimode Plate Reader.

A standard unit (SU) was established using reference serum from a healthy adult to generate a standard curve that was used to normalize samples across experiments. A standard ELISA was used to determine the IgG concentration of this serum sample. The sample was then serially diluted to create a standard curve for each experiment. We defined one SU as the optical density of 0.5 μg/mL IgG of the reference serum. Due to variability in the amount of blood collected on each card, we normalized the microbial ELISA by adding the same amount of IgG (i.e., 50 ng, an amount determined by titration experiments) to each well to determine the relative amount of *Klebsiella*-binding IgG antibodies from each sample.

To determine the amount of IgG binding to common bacterial epitopes, we used *E*. *coli* lipopolysaccharide (LPS) and *Salmonella typhimurium* flagellin. A 100 μl volume of diluted flagellin or LPS (1 μg/mL) was added to each well of a 96-well ELISA plate and incubated overnight at 4°C. The remainder of the ELISA was performed as described above. The secondary anti-LPS and anti-flagellin antibodies were diluted 1:5000.

### Statistical analysis

Nonparametric Mann-Whitney U tests were used to compare *Kleb-*IgG levels and demographic characteristics between neonates with sepsis and controls. A Kruskal-Wallis test was used to compare GA categories between the two groups. Simple linear regression was used to evaluate trends in antibody levels. We conducted an exploratory analysis using logistic regression to evaluate the association of *Kleb-*IgG levels with sepsis while controlling for both GA and days from storage to sample analysis. We conducted an additional exploratory analysis using simple linear regression to explore the association between maternal and neonatal antibody levels among neonates with and without sepsis to evaluate whether antibody transfer differed. Results were considered significant using a two-sided p value (p<0.05).

## Results

### Study participant characteristics

**Fig 1:**
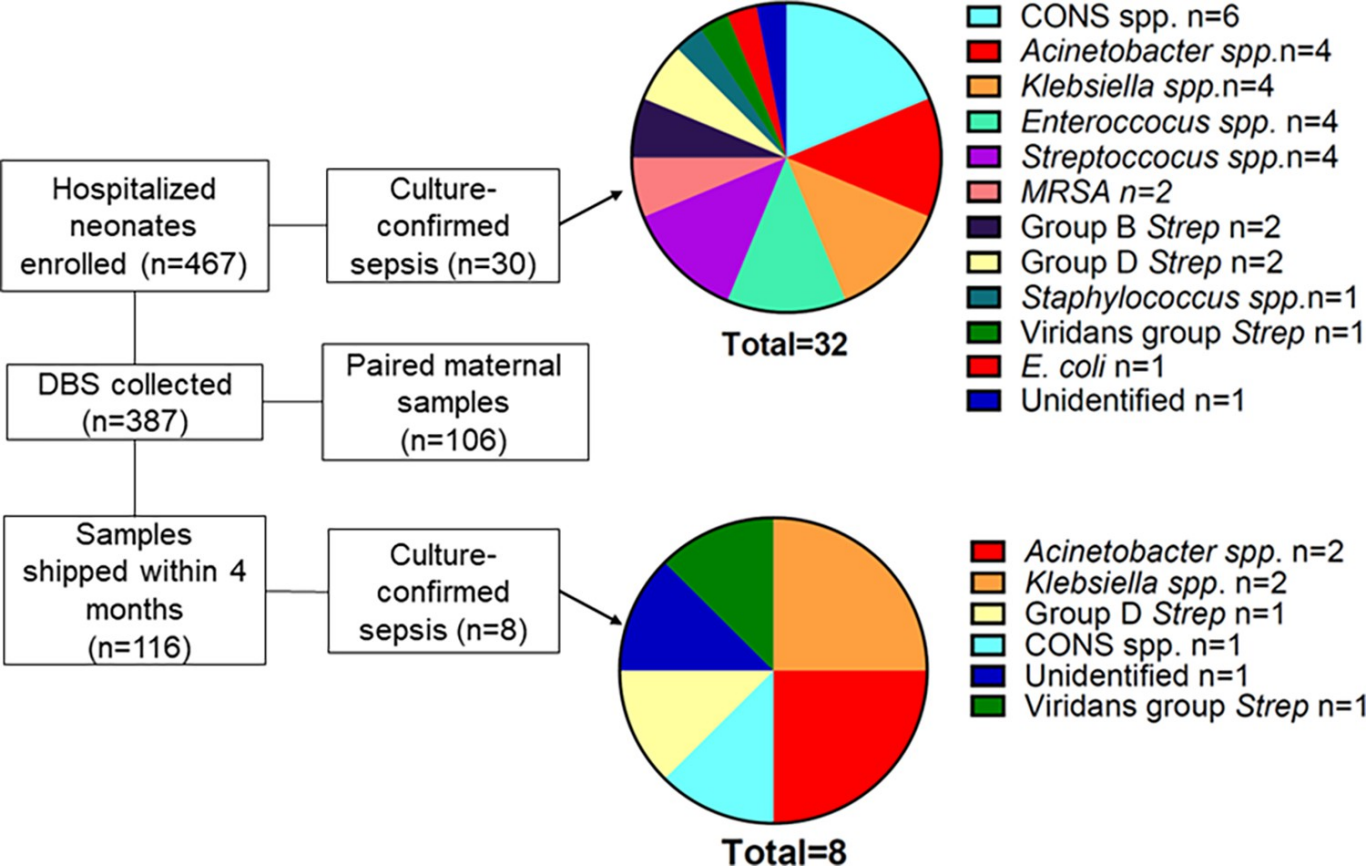
Enrollment numbers, sample numbers, and causes of sepsis in the overall cohort and samples included in this analysis. CoNS = Coagulase-negative *Staphylococcus spp*.; MRSA = methicillin-resistant *Staphylococcus aureus*.

Of the 467 patients enrolled, 30 (6%) experienced bloodstream infections, 116 (24%) had neonatal DBS shipped within 4 months of collection and available for analysis of whom 8 were diagnosed with sepsis **(Fig 1)**. The most common organisms cultured from these neonates with sepsis were *Acinetobacter* spp. and *Klebsiella* spp. which were similar to the etiologies of sepsis in the parent study (**Fig 1**). Cases were matched with controls by GA and sample collection dates. GA remained significantly different between groups, but they were otherwise similar (**Table 1**). Three neonatal samples were missing paired maternal samples and were excluded from the maternal antibody analyses.

**Table 1:** Summary of demographic variables for neonatal sepsis and control groups.

Variable	Sepsis cases (n = 8)	Controls (n = 32)	p value
Female sex, n (%)	4 (50)	14 (44)	0.75
Maternal HIV positive, n (%)	2 (25)	10 (31)	0.73
C-section, n (%)	1 (13)	11 (34)	0.23
Parity, median	1	2	0.56
Gestational age, median	29.5	35.5	**0.01**
Sample collection to analysis, days, median	69	79	0.52

### Klebsiella isolate characterization

We obtained five isolates from the study site including one blood culture isolate from a neonate, three blood culture isolates from adults, and one isolate from an environmental sample. We focused upon the *K*. *pneumoniae* isolate MF391 since that isolate was obtained from a blood culture sample from a hospitalized neonate at the study site in Botswana during the study period. To further characterize this strain, we performed whole genome sequencing using the Illumina NovaSeq platform which provided 10,410,069 reads in 150 bp paired-end format, 129 contigs, N50 = 141,555 and 100% completeness score calculated by CheckM. The genome size is 5,549,078 base pairs, 57.1 GC rich with 96% of the paired reads mapped to the *K*. *pneumoniae* reference genome with an average of 502X coverage and was identified as Sequence Type-26 (ST-26), K type K10, O-type O2afg. It has 3 resistance genes: RmtC (resistance to aminoglycosides), sul1(acquired sulfonamide resistance gene), and blaCTX-M-14 (extended-spectrum beta-lactamase gene).

### Establishing DBS sample integrity

**Fig 2:**
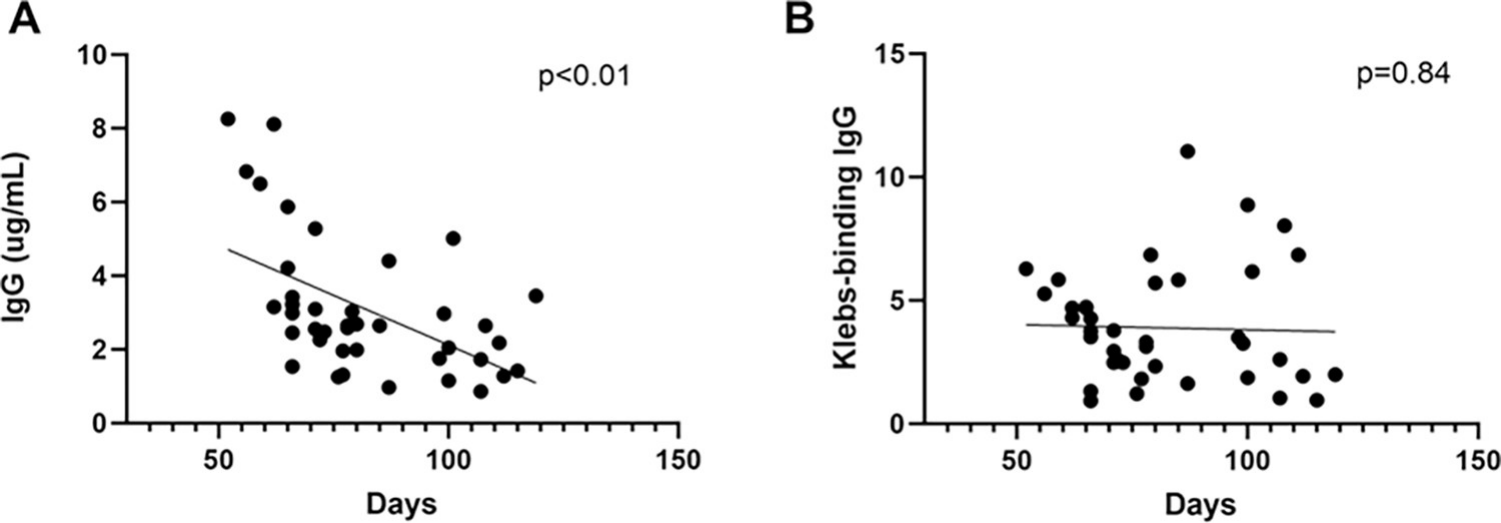
IgG concentration in DBS samples collected within 4 months. **A)** Total IgG concentration recovered from each sample as a function of time interval between sample collection and extraction. **B)** Relative amount of *Kleb-*IgG in each DBS sample, normalized to 0.4ng/μL of total IgG, as a function of time interval between sample collection and extraction. Simple linear regression.

Establishing whether DBS cards stored at room temperature provide valid data is important for studies in settings where immediate storage at -20°C may be unavailable. Prior research demonstrated that most proteins on DBS cards stored at room temperature start degrading after 4 weeks but remain valid for most applications for roughly 6 months, while certain proteins can be detected after much longer periods of storage [26–28]. We, therefore, included the samples with < 4 months of storage at room temperature in this analysis. We evaluated the concentration of total IgG and *Kleb-*IgG from each sample versus time since sample collection to estimate the degree of antibody degradation over time. We recovered lower IgG concentration over time from DBS samples (**Fig 2A).** As such, we normalized the amount of total IgG to 0.4ng /μL in each sample. After normalizing the total IgG concentration in each sample to control for sample degradation, we did not observe a decrease in *Kleb-*IgG binding over time (**Fig 2B**). Degradation of samples during room temperature storage was similar in cases compared to controls **(S2 Fig)**.

As a quality check for both maternal and neonatal sample integrity, we quantified the amount of immunoglobulin A (IgA) recovered from DBS. IgA is not transplacentally transferred nor is it produced by neonates in the first weeks of life [29]. As predicted, maternal DBS samples had detectable serum concentrations of IgA while IgA was not detected in any neonatal samples (**S3 Fig**).

### Neonates with laboratory-confirmed sepsis have lower levels of *Kleb-*IgG

**Fig 3:**
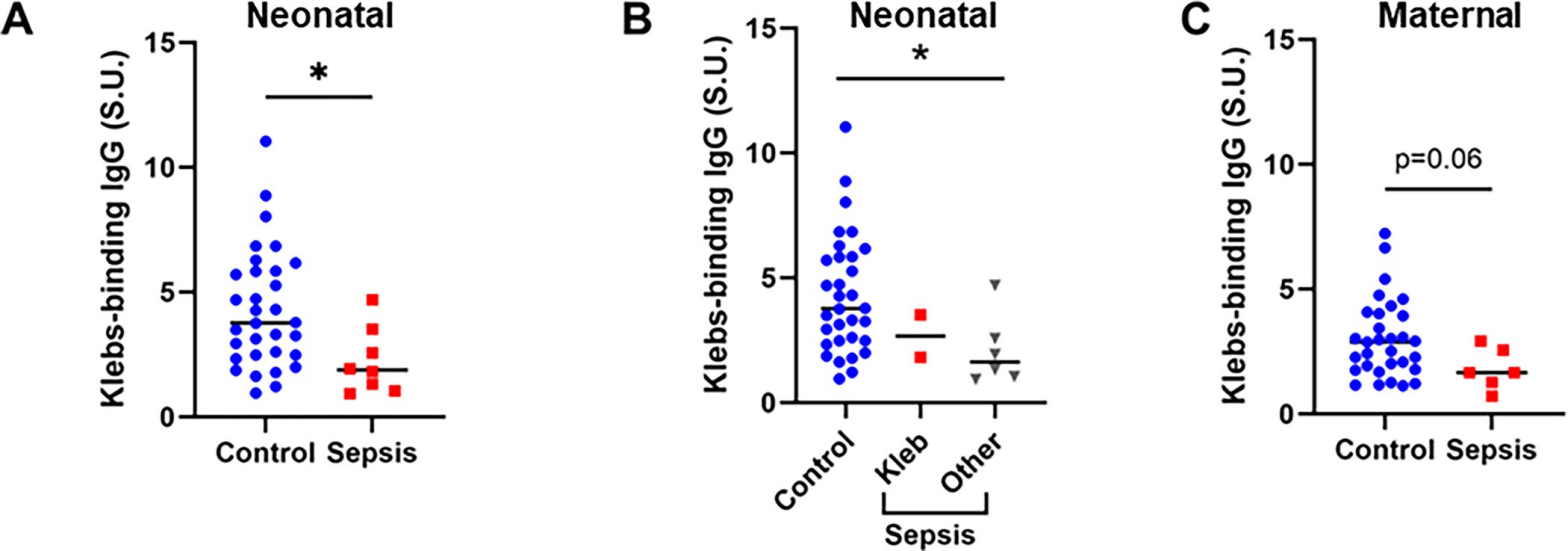
*Kleb-*IgG in neonatal and maternal samples of cases with sepsis compared to controls. **A-B)** *Kleb-*IgG binding in neonates who developed sepsis compared to matched controls. Data were normalized to standard unit (SU), defined as the amount of *Kleb-*IgG in 100 μl of reference serum (healthy adult) with 0.5 μg/mL IgG. Mann-Whitney U test (*p <0.05). **C)** *Kleb-*IgG binding in serum from mothers of neonates who developed sepsis compared to mothers of controls. Kruskal- Wallis test (*p<0.05).

We next investigated whether levels of maternal and neonatal *Kleb-*IgG were associated with increased risk for neonatal sepsis in this cohort of 8 neonates with sepsis and 32 controls without sepsis. We found significantly lower levels of *Kleb-*IgG in neonates who developed sepsis compared to neonates who did not develop sepsis (p = 0.012, **Fig 3A**). *Kleb-*IgG levels were lower in neonates with sepsis regardless of etiology of sepsis (p = 0.04) and not significantly different in samples from neonates with sepsis due to *Klebsiella* compared to other pathogens (**Fig 3B**). Since neonatal IgG concentration and specificities reflect maternal serum IgG, we compared the *Kleb-*IgG levels in samples from mothers of neonates with and without sepsis; samples from mothers of neonates who developed sepsis tended to have less *Kleb-*IgG compared to mothers of controls although the association did not reach statistical significance (p = 0.06, **Fig 3C**). Overall, neonatal and maternal *Kleb-*IgG levels were correlated (r^2^ = 0.27, p<0.01) with a median transfer ratio (neonatal *Kleb*-IgG/maternal *Kleb*-IgG) of 1.3 (**S4 Fig**). In an exploratory analysis, we unexpectedly observed a trend towards an inverse relationship between neonatal and maternal *Kleb-*IgG in dyad samples from patients with sepsis (r^2^ = 0.24, p = 0.32) while a positive association was apparent between neonatal and maternal *Kleb-*IgG levels in those without sepsis (r^2^ = 0.25 p<0.01) **(S5 Fig)**.

The association of low *Kleb-*IgG levels with risk for neonatal sepsis from *Klebsiella* or other bacteria (**Fig 3B**) may suggest that *Kleb-*IgG binding is non-specific and may offer protection through cross-reactivity with common bacterial epitopes. Recent murine studies demonstrated that serum IgG antibodies that bind to commensal microbes protect against bacteremia from pathogenic bacteria [30, 31]. LPS is the most abundant surface molecule in most Gram-negative bacteria, and anti-LPS IgG antibodies can bind to LPS on a variety of microbes [32]. Low levels of serum anti-LPS antibodies are associated with an increased risk of developing sepsis [33]. Similarly, flagellin is a virulence factor present on many bacteria and a common IgG-targeted epitope. Thus, we evaluated the degree to which IgG from each sample binds to LPS or flagellin. We found similar levels of anti-LPS and anti-flagellin IgG antibodies in neonates with sepsis compared to controls (**S6 Fig**). There was no association of *Kleb-*IgG levels with other potential confounders including sex, parity, maternal HIV status, or delivery mode (**S7 Fig**).

### Low *Kleb-*IgG levels are associated with neonatal sepsis across gestational ages

**Fig 4:**
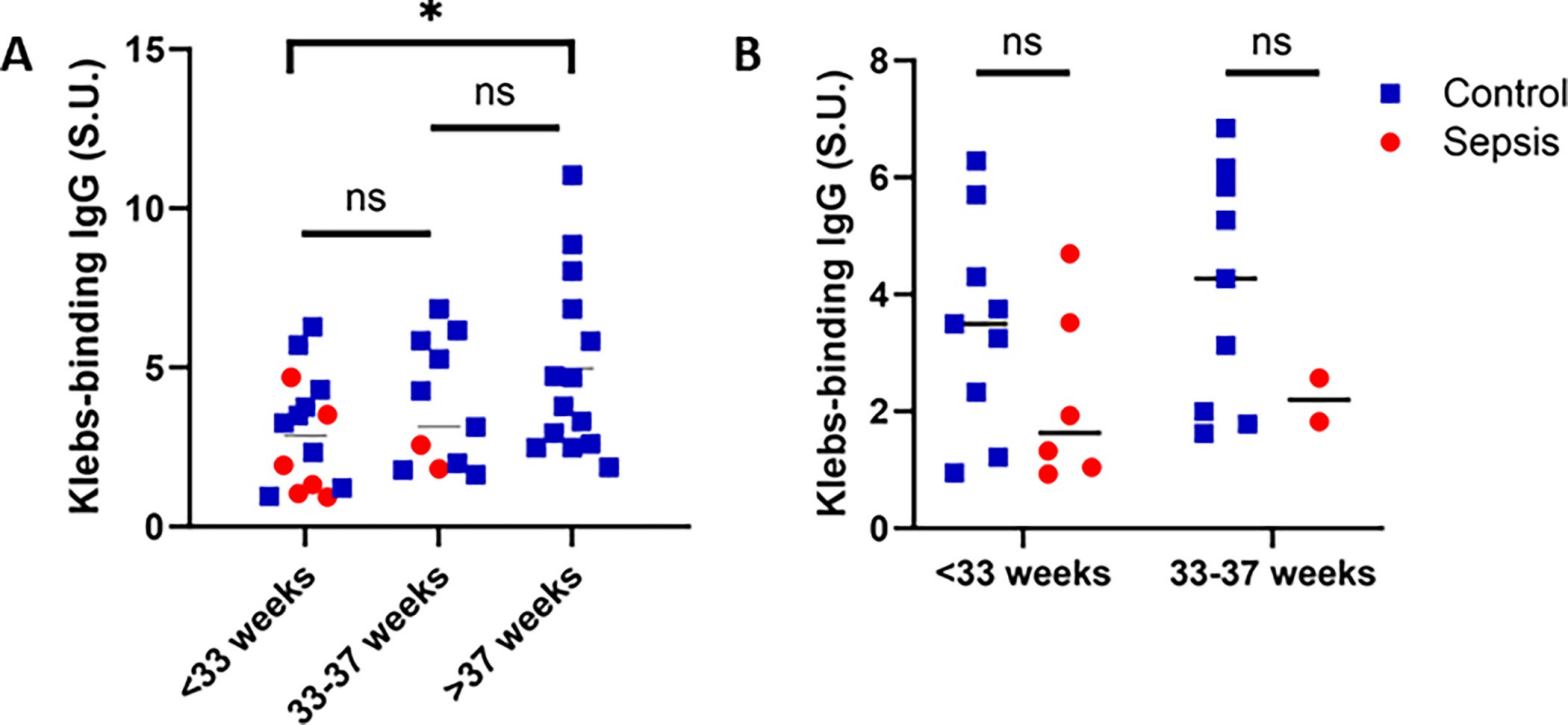
*Kleb-*IgG level by gestational age. **A)** Comparison of *Kleb-*IgG binding in neonates stratified by gestational age. Kruskal-Wallis and Mann-Whitney U test (*p<0.05). **B)** Comparison of *Kleb-*IgG binding in neonates with sepsis compared to controls, stratified by gestational age. Mann-Whitney U test.

Given the differences in median gestational age in controls and sepsis cases, we performed a stratified analysis and found that neonates born at <33 weeks have significantly lower *Kleb-*IgG levels compared to neonates born at ≥37 weeks (p = 0.03) (**Fig 4A**). We evaluated this association in cases compared to controls and found no statistically significant difference in *Kleb-*IgG levels in premature neonates with sepsis compared to controls, yet a trend towards lower levels was noted in samples from neonates born at <33 weeks and between 33–37 weeks (**Fig 4B**). We conducted a sensitivity analysis evaluating the relationship of *Kleb*-IgG by days since sample collection for each GA category and found no difference. In an exploratory analysis, *Kleb-*IgG was associated with a decreased odds ratio (OR) of neonatal sepsis when adjusting for gestational age and days since sample collection (aOR 0.49, 95% CI [0.18, 0.93]). These analyses provide evidence that low *Kleb*-IgG levels are associated with neonatal sepsis across gestational ages.

## Discussion

We report an association between the level of neonatal and maternal serum IgG that bind to *K*. *pneumoniae* and all-cause laboratory-confirmed neonatal sepsis in a cohort of hospitalized neonates in Botswana. Using an ELISA to detect serum antibody reactivity to a *K*. *pneumoniae* strain isolated from a neonate with bacteremia from Gaborone, Botswana, we found that neonates who developed sepsis have lower levels of *Kleb-*IgG compared to controls when adjusting for gestational age and days of sample storage.

*K*. *pneumoniae* was historically the predominant isolate in blood cultures at this site, [7] leading to the development of an ELISA against a *K*. *pneumoniae* isolate from this NICU. During enrollment, the epidemiology of neonatal sepsis shifted from predominantly *Klebsiella* spp. to a more diverse set of microbes (**Fig 1**). Remarkably, neonates with sepsis due to non-*Klebsiella* bacteria also have lower *Kleb-*IgG compared to controls suggesting that *Kleb-*IgG might provide cross-protection against common bacterial epitopes. However, neither anti-LPS nor anti-flagellin IgG antibody levels differed between neonates with sepsis compared to controls, which argues against either being an important cross-reactive microbial epitope in this cohort. These findings suggest the possibility that cross-reactivity to other common epitopes or that multiple epitopes function together to provide protection from neonatal sepsis from a range of pathogens.

By standardizing the total amount of IgG analyzed from each DBS sample, we expected to recover similar levels of *Kleb-*IgG across gestational ages, as we assumed that total IgG and pathogen-specific IgG would cross the placenta at the same rate. However, *Kleb-*IgG levels were lower in preterm neonates which may result from colonization with *Klebsiella* spp. later in pregnancy or increased production of anti-commensal antibodies later in gestation. *Klebsiella* antibodies may vary among pregnant people due to prior infections and differential abundance in the microbiome based on exposure, antibiotics, and other factors [34]. Similarly, Stach *et al* reported increased *Kleb-*IgG with increasing birthweight, a variable that is often colinear with gestational age [35]. Interestingly, another study focusing on pathogen-specific antibodies found no difference in anti-*Klebsiella* IgG by gestational age category [36]. Neither of these studies reported sepsis as an outcome.

Although there were lower levels of *Kleb-*IgG in preterm neonates in our study, the risk of sepsis remained significant when adjusting for GA and days since sample collection indicating that prematurity alone does not explain the association between lower *Kleb-*IgG levels and neonatal sepsis. Since the rate of sample degradation was similar between cases and controls, this is unlikely to account for the association (**S2 Fig**). In an exploratory analysis, we noted a positive linear correlation of maternal and neonatal *Kleb-*IgG among the whole cohort regardless of gestational age, which has been reported previously [36]. However, when we separated cases and controls, we noted a non-statistically significant trend towards an inverse correlation in dyads with sepsis and a statistically significant positive correlation in control dyads which may indicate different rates of placental transfer (**S5 Fig**). Many factors are known to influence transplacental antibody transfer including parity, history of infections, timing of vaccination and placental insufficiency [13, 37]. Since data on placental health, prior infections and maternal hypertension were not available in this cohort, we are unable to analyze these variables.

Our study is limited by sample size and results should be interpreted cautiously. DBS were collected as part of a larger study evaluating hospital exposures and the risk of neonatal sepsis, and some samples were unusable to do slower transit time to the laboratory leading to sample degradation. The sample size is thus limited in this understudied population, yet these results remain helpful for future research directions. Another limitation is that this association between *Kleb*-IgG levels and neonatal sepsis may not be a direct effect of the *Kleb*-IgG binding to pathogens; these antibodies may provide a biomarker of risk of sepsis through decrease levels of antibodies against other pathogens as well, or another mechanism entirely.

Our study highlights the benefits of using DBS for sample collection. Compared to traditional blood tests, DBS can be obtained via minimally invasive methods (heel stick vs. venipuncture), requires a minimal blood volume, remains stable when stored at room temperature for up to 4 months, can be transported easily with minimal risk for contamination, and is an efficient and feasible method to acquire serum samples for a variety of assays [38, 39]. However, there are limitations including variation in collection volume and sample degradation which unfortunately limited the sample size in this study.

## Conclusions

We propose that maternal IgG antibodies against *K*. *pneumoniae* transferred *in utero* may be associated with a decreased risk of sepsis from diverse pathogens. This supports the ongoing development of vaccination strategies during pregnancy to increase pathogen-binding IgG levels in neonates. To protect preterm neonates, a population uniquely at risk for sepsis, further studies are needed to explore mechanisms of efficient transfer of protective maternal antibodies, perhaps capitalizing on functionally enhanced antibodies transferred earlier in pregnancy [40].

## Supporting information

S1 Checklist Inclusivity in global research. (DOCX)

S1 Fig Relative absorbance of IgG from six dried blood spot and one serum sample for five Klebsiella isolates and lipopolysaccharide. A. Sorted by isolate B. Sorted by sample.
(TIF)

S2 Fig Immunoglobulin levels over time in cases compared to controls. **A)** Total IgG concentration recovered from each sample as a function of time interval between sample collection and extraction in sepsis cases compared to controls. **B)** Relative amount of *Kleb-*IgG in each DBS sample as a function of time interval between sample collection and extraction. Standard Unit (SU). Slopes were compared by analysis of covariance.
(TIF)

S3 Fig Levels of IgA in maternal and neonatal DBS samples. IgA level was determined using a standard human ELISA from randomly selected paired maternal and neonatal samples. Mann-Whitney U test. **p<0.01.
(TIF)

S4 Fig Dyad antibody levels. A. Maternal and neonatal dyad total IgG levels. B. Maternal and neonatal *Kleb*-IgG levels.
(TIF)

S5 Fig Correlation between maternal *Kleb-*IgG and neonatal *Kleb-*IgG levels. **A)** *Kleb-*IgG levels for mother-neonate dyads with sepsis, simple linear regression, r^2^ = 0.24, p = 0.32. **B)** *Kleb-*IgG levels for mother-neonate dyads without sepsis, simple linear regression, r^2^ = 0.25, p<0.01.
(TIF)

S6 Fig Anti-LPS IgG and anti-flagellin IgG in neonates with and without sepsis. **A)** ELISA comparing anti-LPS IgG in neonates with sepsis vs. controls. All units are normalized to the standard unit, defined as the amount of anti-LPS IgG in 50 ng of reference adult serum. **B)** ELISA comparing anti-flagellin IgG in neonates with sepsis vs. controls. Mann-Whitney U test.
(TIF)

S7 Fig *Kleb-*IgG level by maternal HIV status, neonatal sex, delivery mode, and maternal parity. **A-D)** *Kleb-*IgG binding comparison by the indicated clinical variables. Mann-Whitney U test or Kruskal-Wallis test.
(TIF)

S1 Data Data for the manuscript. (XLSX)
