# Supplementary figures and images for: Maternal and neonatal IgG against Klebsiella pneumoniae are associated with lower risk of neonatal sepsis: A case-control study of hospitalized neonates in Botswana
Source: PLOS Glob Public Health. 2024 Dec 5;4(12):e0003350. doi: 10.1371/journal.pgph.0003350 (PMC11620667; doi:10.1371/journal.pgph.0003350)

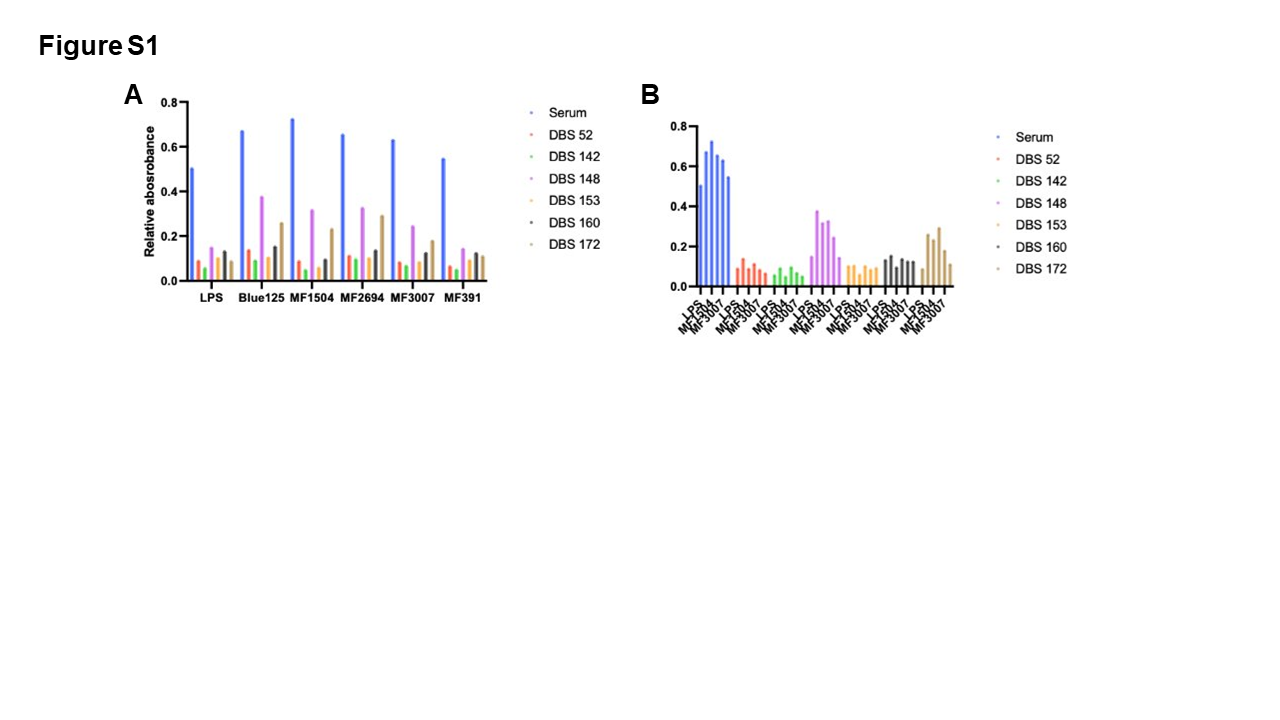

Supplement: S1 Fig — A. Sorted by isolate B. Sorted by sample. (TIF) [file pgph.0003350.s002.tif]

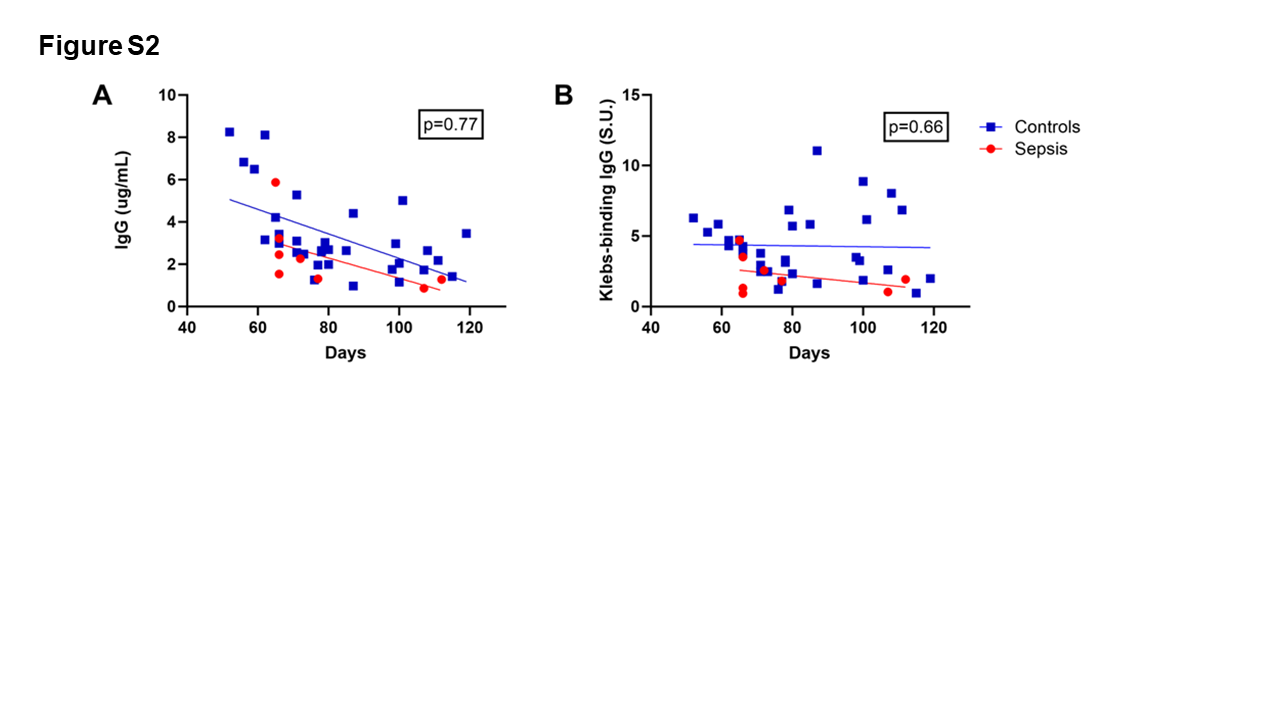

Supplement: S2 Fig — A) Total IgG concentration recovered from each sample as a function of time interval between sample collection and extraction in sepsis cases compared to controls. B) Relative amount of Kleb-IgG in each DBS sample as a function of time interval between sample collection and extraction. Standard Unit (SU). Slopes were compared by analysis of covariance. (TIF) [file pgph.0003350.s003.tif]

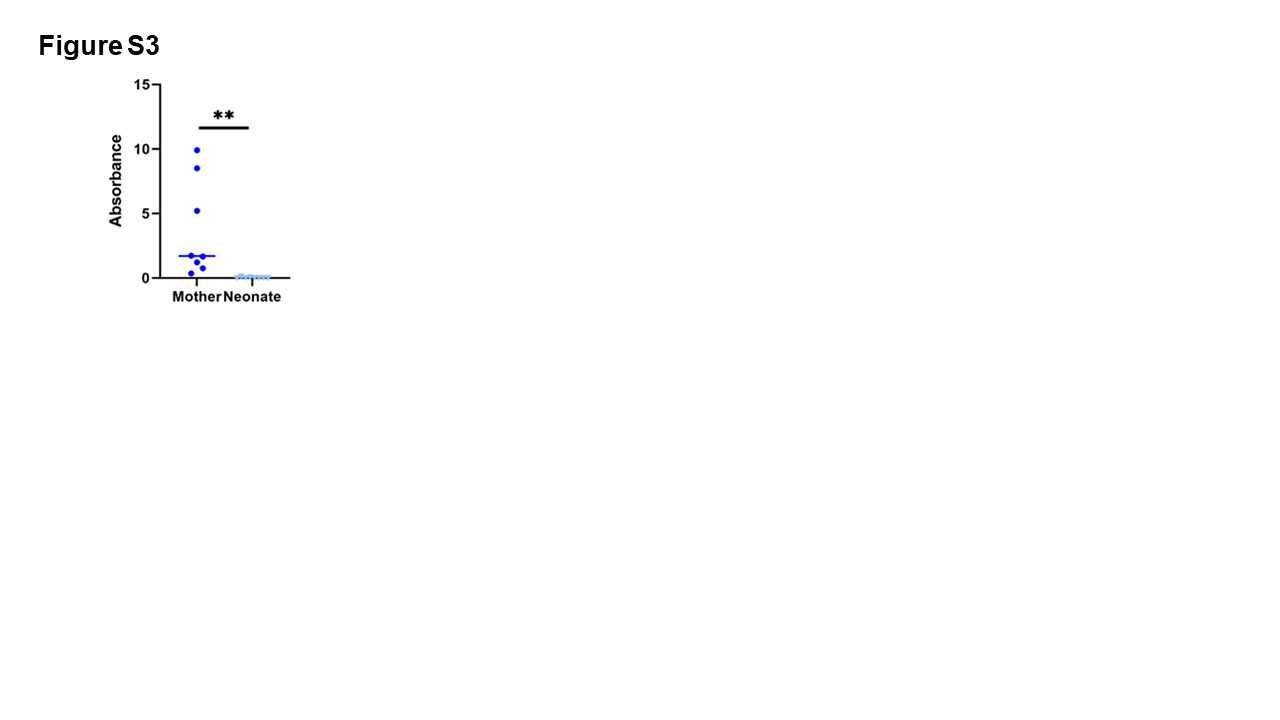

Supplement: S3 Fig — IgA level was determined using a standard human ELISA from randomly selected paired maternal and neonatal samples. Mann-Whitney U test. **p<0.01. (TIF) [file pgph.0003350.s004.tif]

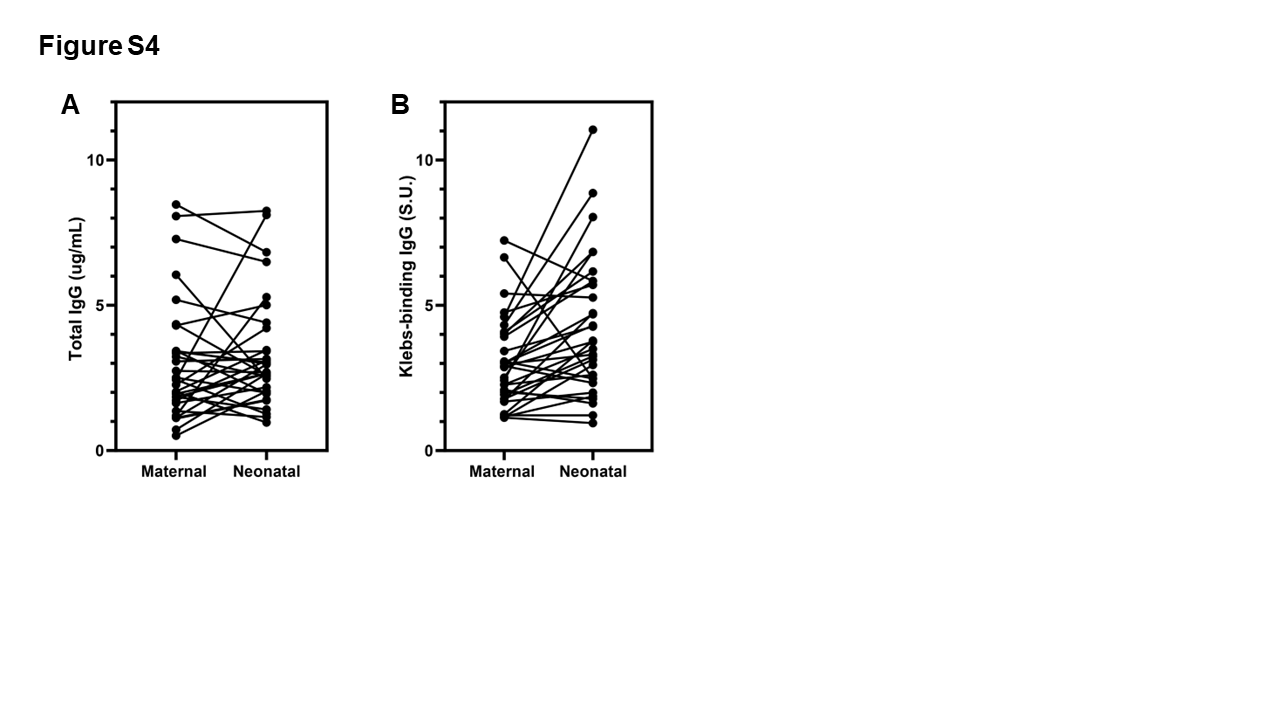

Supplement: S4 Fig — A. Maternal and neonatal dyad total IgG levels. B. Maternal and neonatal Kleb-IgG levels. (TIF) [file pgph.0003350.s005.tif]

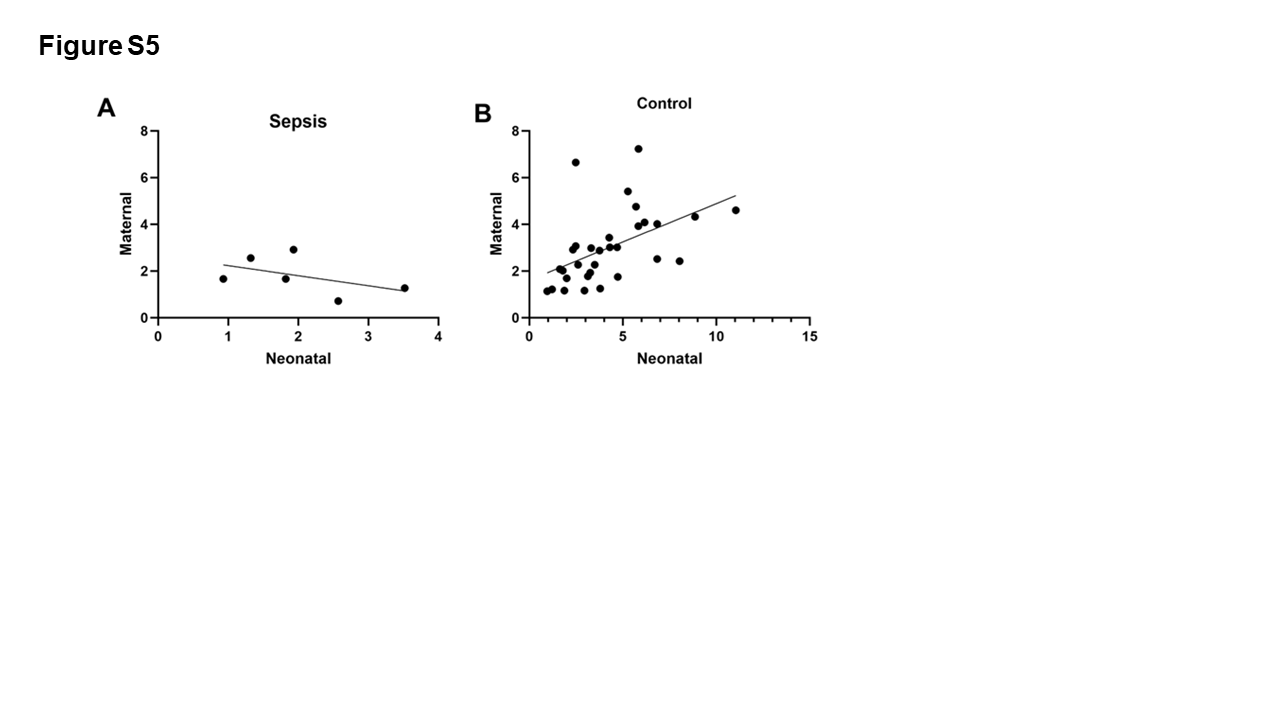

Supplement: S5 Fig — A) Kleb-IgG levels for mother-neonate dyads with sepsis, simple linear regression, r2 = 0.24, p = 0.32. B) Kleb-IgG levels for mother-neonate dyads without sepsis, simple linear regression, r2 = 0.25, p<0.01. (TIF) [file pgph.0003350.s006.tif]

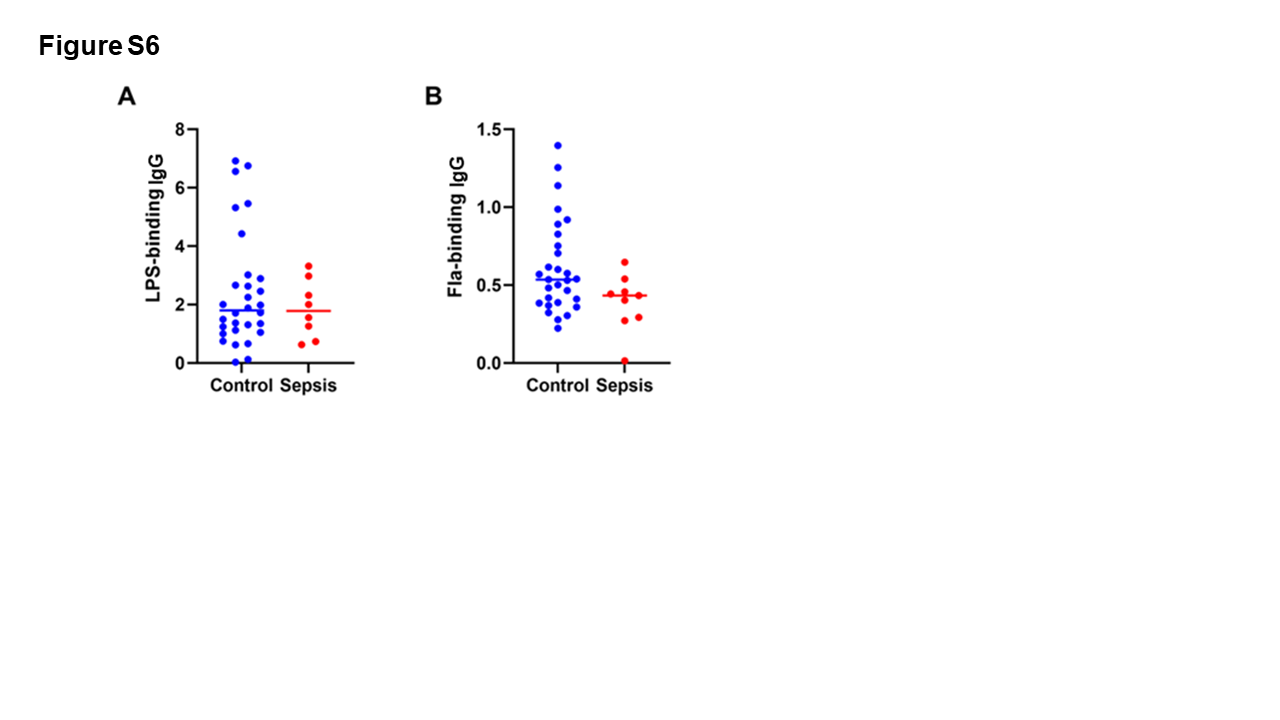

Supplement: S6 Fig — A) ELISA comparing anti-LPS IgG in neonates with sepsis vs. controls. All units are normalized to the standard unit, defined as the amount of anti-LPS IgG in 50 ng of reference adult serum. B) ELISA comparing anti-flagellin IgG in neonates with sepsis vs. controls. Mann-Whitney U test. (TIF) [file pgph.0003350.s007.tif]

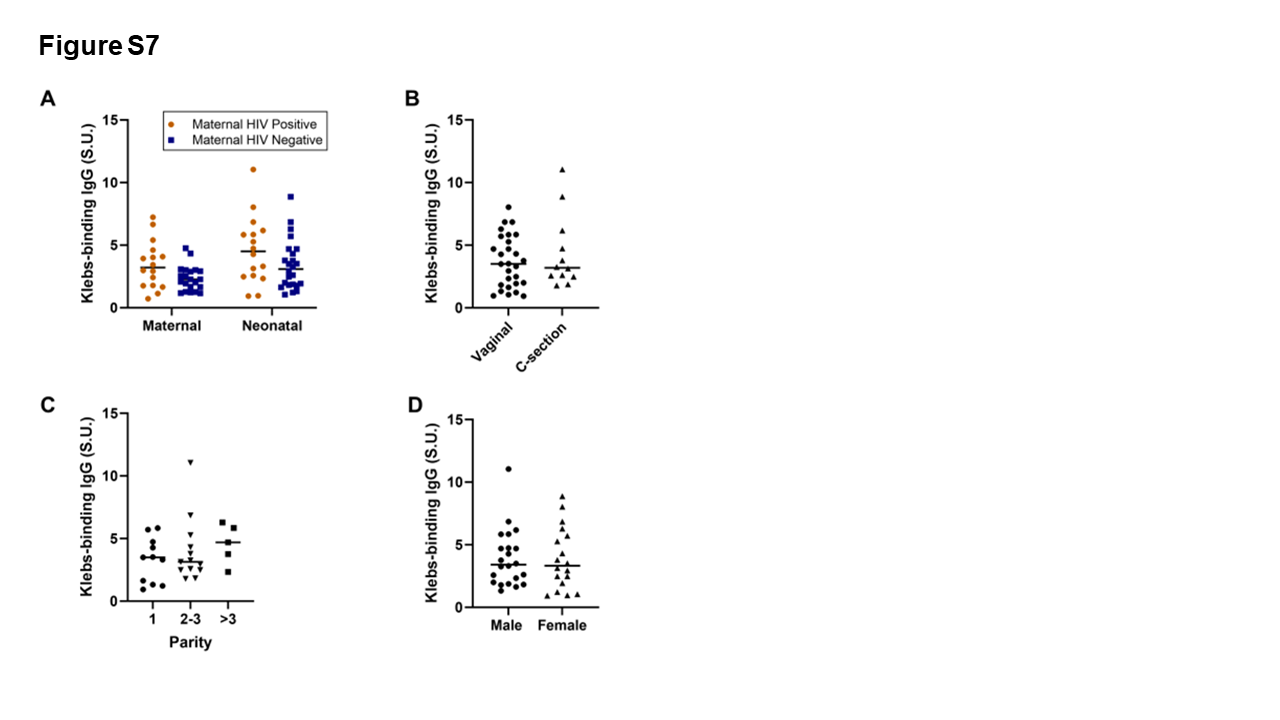

Supplement: S7 Fig — A-D) Kleb-IgG binding comparison by the indicated clinical variables. Mann-Whitney U test or Kruskal-Wallis test. (TIF) [file pgph.0003350.s008.tif]
